# Walrus optimizer-based optimal fractional order PID control for performance enhancement of offshore wind farms

**DOI:** 10.1038/s41598-024-67581-x

**Published:** 2024-07-31

**Authors:** Mohamed A. M. Shaheen, Hany M. Hasanien, S. F. Mekhamer, Hossam E. A. Talaat

**Affiliations:** 1 Electrical Power and Machines Department, Faculty of Engineering, Ain Shams University, Cairo, 11517 Egypt; 2 Electrical Engineering Department, Faculty of Engineering and Technology, Future University in Egypt, Cairo, 11835 Egypt

**Keywords:** WaOA, High voltage direct current, Voltage source converter, Offshore wind farms, Electrical and electronic engineering, Energy infrastructure

## Abstract

Offshore wind farms (OWFs) play a crucial role in producing renewable energy in modern electrical power systems. However, to ensure that these facilities operate smoothly, they require robust control systems. As a result, this paper employed the newly developed Walrus Optimization algorithm (WaOA) to optimize the design parameters of fractional-order proportional-integral-derivative (FOPID) controllers in the power electronic interface circuits of the studied wind energy conversion system (WECS). In contrast to conventional optimization techniques like GA and PSO, the suggested approach proves more effective. The paper validates the WaOA application in optimizing FOPID controllers within a WECS comprising two, onshore and offshore, VSC stations at the two ends of an HVDC transmission system connecting OWFs to the mainland. The study shows that the WaOA outperforms GA and PSO, improving system stability and enabling quick recovery after disturbances. The study carried out using MATLAB/Simulink highlights the significance of newly recently introduced optimization techniques to ensure efficient and reliable operation of offshore wind energy systems, thereby expediting the transition to sustainable energy sources.

## Introduction

Sustainable and environmentally friendly energy sources should be used due to the growing worldwide demand for energy and the need for action for solutions to climate change^1^. After being the primary energy source for many years, fossil fuels have come under criticism for their negative impacts on the environment including pollution and greenhouse gas emissions^2,3^. Due to its ability to provide a clean and sustainable alternative for grid integration as well as power generation, renewable energy sources like wind and solar power are becoming more and more popular worldwide^4,5^. Efficient integration of variable and intermittent energy sources into current power networks is an important challenge in the shift to renewable energy^6,7^.

Wind energy is an important renewable energy source that should replace fossil fuels in the electrical power generation field^8^. Wind power is considered a green source of energy, offering a sustainable solution to the energy demands^9^. There are two primary ways to use wind energy: onshore and offshore^10^. Onshore wind farms are more common now since they are easier to reach and have cheaper establishing costs^11^. Nevertheless, offshore wind energy has enormous potential, considering the better wind conditions at sea^12^. Offshore wind farms (OWFs) provide a remarkable growth in electrical power generation while being more complicated and expensive to generate^13^. The transmission of electrical power from these offshore sites to the mainland is achieved through HVDC systems.

High Voltage Direct Current (HVDC) systems are a major development in the field of electrical transmission systems, offering a number of advantages that highlight their importance^14^. One advantage of HVDC systems is their lower corona loss rates^15^. This lowers energy waste and, in turn, the carbon footprint, making HVDC systems more environmentally sustainable. Moreover, HVDC systems are efficient at transferring power, enabling the effective and low-loss transfer of electrical power over extended distances^16^. This capacity is essential for improving the performance and reliability of the grid, particularly in cases when the integration of renewable energy sources that are remote from centers of consumption is involved. HVDC systems can assist in lowering the expenses related to the transmission of electrical power by providing increased control over power flows and providing lower transmission losses^16,17^. As a result, the use of HVDC technology means a more efficient, and sustainable future for the energy systems^18^.

In the field of wind power generation, Permanent Magnet Synchronous Generators (PMSGs) and Doubly Fed Induction Generators (DFIGs) are the two most commonly used generator types^19^. Both have unique benefits in terms of efficiency and adaptability. PMSG is a preferred choice for integration with Variable Speed Wind Turbines (VSWT) within Wind Energy Conversion Systems (WECS) due to their remarkable performance^20^. PMSGs are highly efficient, requiring less maintenance due to their brushless design^21^. Furthermore, because they can operate at different speeds, they can optimally capture energy from wind^22^, which makes them suitable for VSWT applications^23^. The integration of PMSGs into WECS enhances the overall efficiency and reliability of wind energy production. This dynamic field of research continues to grow, with ongoing research and development aimed at maximizing the potential of wind energy for the future energy mix. There are difficulties in integrating OWFs into the power grid, especially when handling different fault scenarios that might affect the reliability of bulk power transmission^24^. An advanced controller architecture is required for grid-connected HVDC systems to guarantee dependable and effective power flow. The core of this integration is the Voltage Source Converter (VSC), and key topologies include the cascaded H-bridge^25^, flying capacitor^26^, and neutral point clamping^27^. Every architecture has a unique set of advantages and disadvantages.

Several control strategies are used to operate VSC stations to guarantee dependability, and stability. To achieve small voltage regulation, good power quality, and active power regulation at the inverter station, the research^28^ presents a nonlinear observer-based output feedback control approach for HVDC systems utilizing the backstepping design technique. The paper^29^ introduces modifications to Virtual Synchronous Generator control for converter stations to enhance frequency stability in HVDC systems. A model predictive control (MPC) technique is proposed in^30^, to manage the dynamic performance of an interconnected HVDC system with superconducting magnetic energy storage, and its effectiveness and robustness are demonstrated through MATLAB simulations under various disturbances. Moreover, a broad range of techniques have been developed to address challenging optimization problems efficiently. The article^31^ proposes a new MPPT algorithm based on parabolic prediction techniques for wind turbines. The PPT-MPPT aims to improve wind power extraction by estimating the wind turbine's power curve and continuously adjusting its tracking under varying wind speeds. Reference^32^ proposes a new hybrid optimization algorithm combining Sine Cosine Algorithm and Transient Search Optimization for designing power converter controls in wind turbine systems. Reference^33^ proposes the Sine Cosine Algorithm to optimize the gains of PI controllers in a VSWT system. The algorithm aims to maximize system performance by adjusting the controller settings for both the machine-side and the grid-side converters.

Introduced in 2024, the Walrus Optimization Algorithm (WaOA) represents a metaheuristic in the field of optimization algorithms, inspired from the behaviors of walruses^34^. This includes their migration patterns, breeding habits, sleeping routines, feeding strategies, gathering activities, and mechanisms for escaping predators. A key component of WaOA's methodology is the modeling of walrus behavior in response to safety and danger signals, which simulates these natural tactics to explore the search space of optimization problems^35^. The WaOA can be a preferable option for resolving complex problems due to its superior convergence speed and efficiency compared to other algorithms. It is recommended to employ the WaOA for further optimization problems.

The Fractional Order Proportional-Integral-Derivative (FOPID) controller demonstrates superior performance compared to conventional Proportional-Integral (PI) controllers in a variety of control system applications^36,37^. The FOPID controller utilizes fractional calculus instead of integer-order calculus used by the PI controller. This allows for greater flexibility and fine-tuning by incorporating additional parameters and the orders of the derivative and integral operations. The recent improvement in the FOPID controller has made it more efficient in handling non-linear dynamics. As a result, the systems controlled by FOPID controllers have smoother and more consistent responses compared to those controlled by conventional PI controllers. Additionally, FOPID controllers lead to quicker settling times and less overshoot. This study aims to analyze the effectiveness of the WaOA in VSC-based HVDC WECS to enhance the performance of FOPID controllers. The paper provides a detailed investigation of the WaOA's output compared to other metaheuristic algorithms for both symmetrical and unsymmetrical fault scenarios, supported by Simulink simulations. The primary focus of this research is to identify how to maximize the benefits of FOPID controllers and improve system performance. This study presents three main contributions. Firstly, it explains how the WaOA is utilized to adjust the gains of the FOPID controller in the VSC station. Secondly, it evaluates the optimized system's reliability in various fault scenarios, highlighting the WaOA's resilience. Finally, the study demonstrates that, in comparison to other optimization strategies, the WaOA can enhance the system's transient performance, as evidenced by improved settling times, reduced peak overshoot, and minimized maximum power undershoot.

The remaining of this paper is divided as follows: The system configuration is presented in Section "Configuration of the studied system", the proposed optimization algorithm is presented in Section "The proposed optimization algorithm", and the FOPID parameters’ gains and simulation results are described in Section "Simulation results". Lastly, Section "Conclusions" concludes the paper mentioning the recommendations for the future work.

## Configuration of the studied system

**Figure 1:**
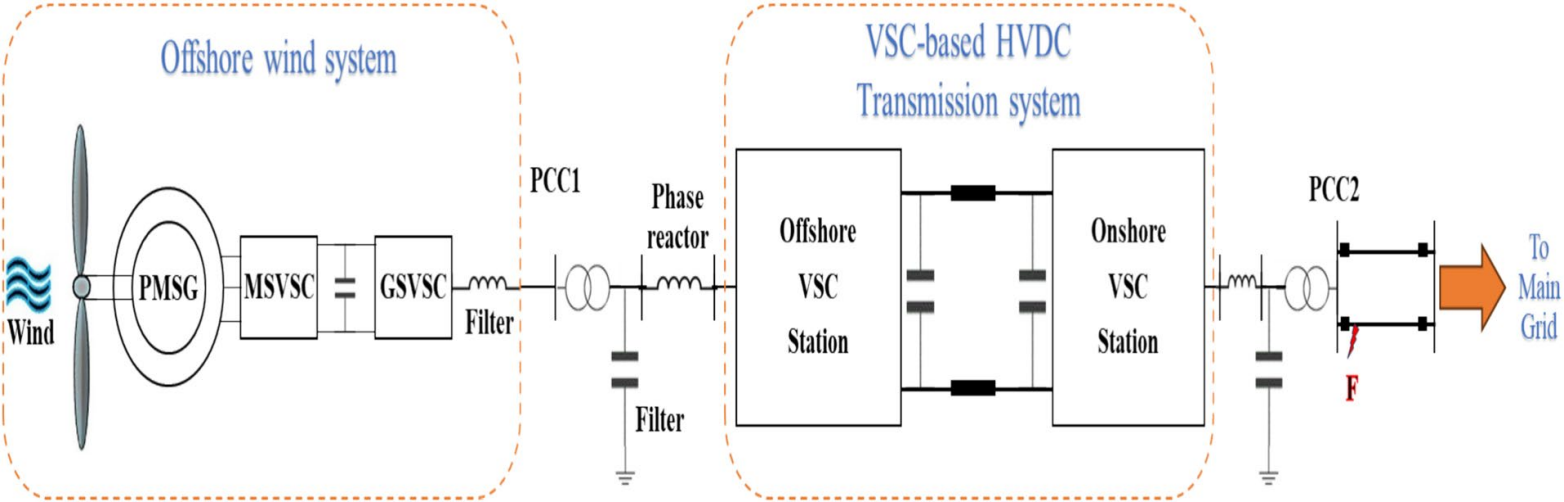
A schematic diagram of the studied system.

The studied system is shown in Fig. 1, which illustrates an OWF with a VSC-based HVDC transmission system. The system is divided into two main sections; (1) Offshore Wind Turbine System which starts with a wind turbine. The wind turbine harnesses the kinetic energy of wind and converts it into mechanical energy. The mechanical energy is then converted into electrical energy using PMSG. The AC power from the PMSG is fed into the first VSC, the machine side voltage source converter (MSVSC). The MSVSC acts as a rectifier. A DC link smoothing capacitor is used to smooth the ripples in the DC power. The DC power is then fed into the second VSC, the grid side voltage source converter (GSVSC). The GSVSC inverts the DC power back into AC power. The two VSCs, MSVSC and GSVSC, are part of the offshore wind turbine system. (2) VSC-based HVDC Transmission System, the second part of the system, includes two VSC stations; offshore VSC station and onshore VSC station. Between them, there is a DC marine cable that is used to transmit the electrical energy from the OWF to the onshore side of the grid. The first VSC station, connected to the offshore wind system, acts as a rectifier, converting the electrical energy from AC to DC before transmission through the marine cable. After the marine cable, the second VSC station acts as an inverter, converting the DC back to AC for the integration with the onshore power grid. Transformer matches the voltage level for the grid and the onshore station.

The PMSG parameters are listed in Table 1. The HVDC and VSC stations parameters values are also listed in Table 2. The DC voltage, active power, reactive power, and AC voltage are all controlled by the converters. The converters include inner and outer control loops. While the external controllers control the power, the internal controllers control the current. These cascaded loops preserve the efficiency and stability of the system. Small-scale wind turbines have been replaced with a massive 150 MW generator that operates at 50 Hz and 690 kV.

**Table 1:** PMSG parameters.

PMSG	Rated value
Rated active power	150 MW
Rated voltage	690 V
Frequency	20 Hz
No. of pole pairs	75
Stator resistance	0.01 p.u
*d*-axis reactance	1 p.u
*q*-axis reactance	0.7 p.u
Flux linkage	1.4 p.u
Inertial	0.5 p.u

**Table 2:** HVDC cable and converter parameters.

Parameter	Rated value
Conductor radius	1.78 cm
Insulator radius	3.70 cm
Copper resistivity	17.24 nΩ m
Permittivity	2.3
Resistance at 20 °C	17.2 µΩ/m
Snubber resistance	5 kΩ
Snubber capacitance	1 µF
Length of HV cable	75 km

### VSC-controlled VSWT-PMSG

The offshore station has two VSCs and a PMSG driven by a wind turbine. ${P}_{m}$ represents the mechanical output power. The following Eq. (1), represents the mathematical model of the turbine^38^:

1 $${P}_{m}=0.5\rho A{C}_{P}(\lambda ,\beta ){V}_{w}^{3}$$

where the speed of the wind is represented by the symbol ‘${V}_{w}$’ in meters per second. The area that the rotor of the wind turbine covers is represented by the letter ‘$A$’. The tip speed ratio is represented by ‘$\lambda$’, while the air density is represented by ‘$\rho$’ in kilograms per cubic meter. The angle of the blade pitch is represented by ‘$\beta$’. The ratio of the available power to the mechanical power is determined by the optimum power coefficient, ${C}_{P}$.

**Figure 2:**
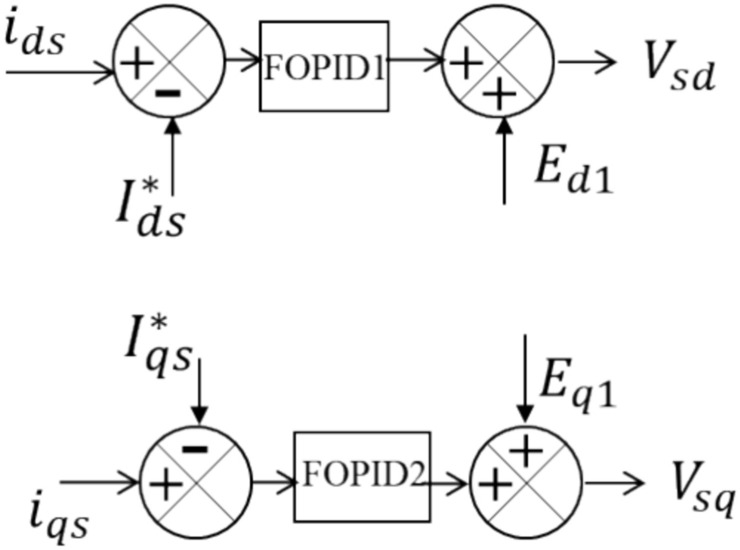
MSVSC controller setup.

The MSVSC, which is connected to the PMSG, converts AC to DC, as shown in Fig. 1. To control the VSWT-PMSG, a cascaded control approach is used. The reference value for the d-axis current component, ${I}_{ds}^{*}$, is set to 0. The q-axis current component is represented concurrently by ${i}_{qs}$, and ${I}_{qs}^{*}$ is its matching reference value. The PMSG's rotating speed and ${I}_{qs}^{*}$ have a straight proportional relationship. ${I}_{qs}^{*}$ impacts the real power reference, P_ref_. Optimizing the amount of power delivered to the DC bus is the main goal of this impact. The values of ${E}_{d1}$ and ${E}_{q1}$ represent the voltage components along the d- and q- axes, respectively. The ${i}_{d}$ and ${i}_{q}$ currents are controlled by the FOPID controllers, whose output is combined with ${E}_{d1}$ and ${E}_{q1}$. This produces the dq components of the stator voltages, also known as ${V}_{sd}$ and ${V}_{sq}$. These components are then utilized to determine the MSVSC gate signals. Figure 2 illustrates the setup of the MSVSC controller.

The converter has been used to control the turbine's speed to maximize output^39^. By modifying the rotor speed, the MPPT controller maximizes the amount of electricity harvested from the wind. The appropriate torque to provide the DC link with the most power is controlled by the q-axis portion of the current^40^. To achieve a unity power factor, the system is operated at zero reactive power by adjusting the d-axis current. Equations (2) and (3) display the stator voltage's dq components^38^.

2 $$ \begin{gathered} V_{sd} = R_{s} I_{sd} + L_{s} \frac{{dI_{sd} }}{dt} + E_{d1} \hfill \\ E_{d1} = {\Omega }L_{s} I_{sq} \hfill \\ \end{gathered} $$

3 $$ \begin{gathered} V_{sq} = R_{s} I_{sq} + L_{s} \frac{{dI_{sq} }}{dt} + E_{q1} \hfill \\ E_{q1} = {\Omega }\left( {\psi_{s} - L_{s} I_{sd} } \right) \hfill \\ \end{gathered} $$

where the generator resistance is ${R}_{s}$. The generator inductance is ${L}_{s}$. ${I}_{sd}$ and ${I}_{sq}$ stand for the d-q axis currents, and $\Omega$ denotes the rotor speed. Magnetic flux linkage is denoted by $\psi$. The q-axis component of the stator flux, ${\psi }_{sq}$, is set to zero since the stator flux is aligned with the reference frame. Because of this alignment, ${\psi }_{s}$ and ${\psi }_{sd}$ are equal. Equations (4) and (5) represent the control for the inner current control loops implemented as FOPID1 and FOPID2 controllers.

4 $${R}_{s}{I}_{sd}+{L}_{s}\frac{d{I}_{sd}}{dt}={k}_{p1}\left({I}_{sd}-{I}_{sd}^{*}\right)+{k}_{i1}{\left(\int \left({I}_{sd}-{I}_{sd}^{*}\right)dt\right)}^{{\alpha }_{1}}+{k}_{d1}\frac{{d}^{{\mu }_{1}}\left({I}_{sd}-{I}_{sd}^{*}\right)}{{dt}^{{\mu }_{1}}}$$

5 $${R}_{s}{I}_{sq}+{L}_{s}\frac{d{I}_{sq}}{dt}={k}_{p2}\left({I}_{sq}-{I}_{sq}^{*}\right)+{k}_{i2}{\left(\int \left({I}_{sq}-{I}_{sq}^{*}\right)dt\right)}^{{\alpha }_{2}}+{k}_{d2}\frac{{d}^{{\mu }_{2}}\left({I}_{sq}-{I}_{sq}^{*}\right)}{{dt}^{{\mu }_{2}}}$$

where ${k}_{p1}$, ${k}_{i1}$, ${k}_{d1}$, is the proportional, integral, and derivative gains for the FOPID1. To fine-tune the response of the FOPID1 controller, the fractional integral term includes an exponent, ${\alpha }_{1}$. The exponent ${\mu }_{1}$ in the derivative term indicates that the derivative is fractional, enabling more accurate controller behavior. Similarly, ${k}_{p2}$, ${k}_{i2}$, ${k}_{d2}$, ${\alpha }_{2}$, and ${\mu }_{2}$ are the gains and the exponents of the controller FOPID2, respectively.

**Figure 3:**
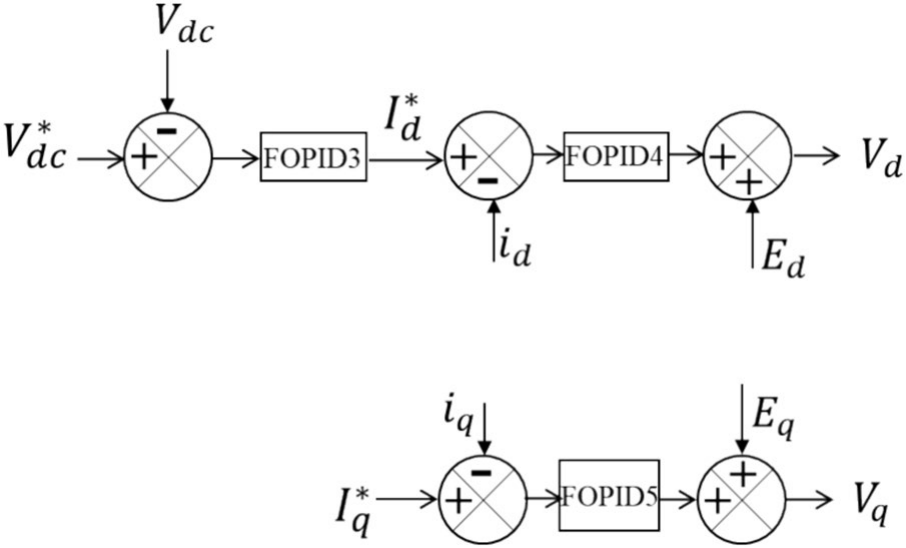
GSVSC controller setup.

A GSVSC connected to the AC bus is also part of the system. The GSVSC is responsible for controlling the system's reactive power, or ${i}_{q}$, and maintaining the DC bus voltage, or ${V}_{dc}$, at a specific setpoint, ${V}_{dc}^{*}$. Equations (6) and (7) determine the RMS voltage of the ${V}_{d}$ and ${V}_{q}$, the d- and q-axis components of the voltage across the GSVSC^38^. Figure 3 shows the setup of the GSVSC controller. ${I}_{d}^{*}$, which is compared with ${I}_{d}$, is obtained by feeding the controller FOPID3 with the difference between ${V}_{dc}^{*}$ and ${V}_{dc}$. The error signal is then sent to the FOPID4 controller, whose output is ${V}_{d}$. ${I}_{q}^{*}$ is compared to ${i}_{q}$ for the reactive power regulation, and the error is handled by the controller FOPID5, whose output is ${V}_{q}$. To summarize the GSVSC control scheme, the d-axis controls the DC link voltage, and the q-axis regulates the reactive power. This scheme uses decoupled control.

6 $$ \begin{gathered} V_{d} = R_{1} i_{d} + L_{1} \frac{{di_{d} }}{dt} + E_{d} \hfill \\ E_{d} = V_{pcc1,d} - \omega L_{1} i_{q} \hfill \\ \end{gathered} $$

7 $$ \begin{gathered} V_{q} = R_{1} i_{q} + L_{1} \frac{{di_{q} }}{dt} + E_{q} \hfill \\ E_{q} = V_{pcc1,q} + \omega L_{1} i_{d} \hfill \\ \end{gathered} $$

The voltage at the PCC1 is made up of two components along the d- and q-axes, which are represented by ${V}_{pcc1, d}$ and ${V}_{pcc1, q}$. The current flowing across the GSVSC also has two components along the d- and q-axes, which are referred to as ${i}_{d}$ and ${i}_{q}$. The series filter has two parameters, the resistance ${R}_{1}$ and the inductance ${L}_{1}$. The angular velocity of the AC side voltage is denoted by $\omega$.

The outer DC voltage circuit controller of FOPID3 has gains represented by ${k}_{p3}$, ${k}_{i3}$, ${k}_{d3}$, ${\alpha }_{3}$, and ${\mu }_{3}$ in Eq. (10). In contrast, the inner circuit's FOPID4 and FOPID5 parameters have the following gains: ${k}_{p4}$, ${k}_{i4}$, ${k}_{d4}$, ${\alpha }_{4}$, ${\mu }_{4}$, ${k}_{p5}$, ${k}_{i5}$, ${k}_{d5}$, ${\alpha }_{5}$, and ${\mu }_{5}$, as shown in Eqs. (8) and (9).

8 $${R}_{1}{i}_{d}+{L}_{1}\frac{d{i}_{d}}{dt}={k}_{p4}\left({I}_{d}^{*}-{i}_{d}\right)+{k}_{i4}{\left(\int \left({I}_{sd}-{I}_{sd}^{*}\right)dt\right)}^{{\alpha }_{4}}+{k}_{d4}\frac{{d}^{{\mu }_{4}}\left({I}_{sd}-{I}_{sd}^{*}\right)}{{dt}^{{\mu }_{4}}}$$

9 $${R}_{1}{i}_{q}+{L}_{1}\frac{d{i}_{q}}{dt}={k}_{p5}\left({I}_{q}^{*}-{i}_{q}\right)+{k}_{i5}{\left(\int \left({I}_{sq}-{I}_{sq}^{*}\right)dt\right)}^{{\alpha }_{5}}+{k}_{d5}\frac{{d}^{{\mu }_{5}}\left({I}_{sq}-{I}_{sq}^{*}\right)}{{dt}^{{\mu }_{5}}}$$

10 $${i}_{dref}={k}_{p3}\left({V}_{dc}^{*}-{V}_{dc}\right)+{k}_{i3}{\left(\int \left({V}_{dc}^{*}-{V}_{dc}\right)dt\right)}^{{\alpha }_{3}}+{k}_{d3}\frac{{d}^{{\mu }_{3}}\left({V}_{dc}^{*}-{V}_{dc}\right)}{{dt}^{{\mu }_{3}}}$$

### VSC-based HVDC transmission

**Figure 4:**
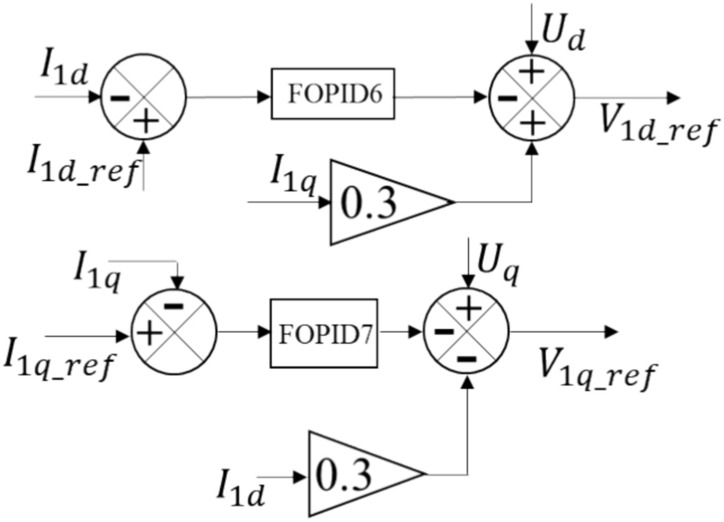
Inner current control setup.

**Figure 5:**
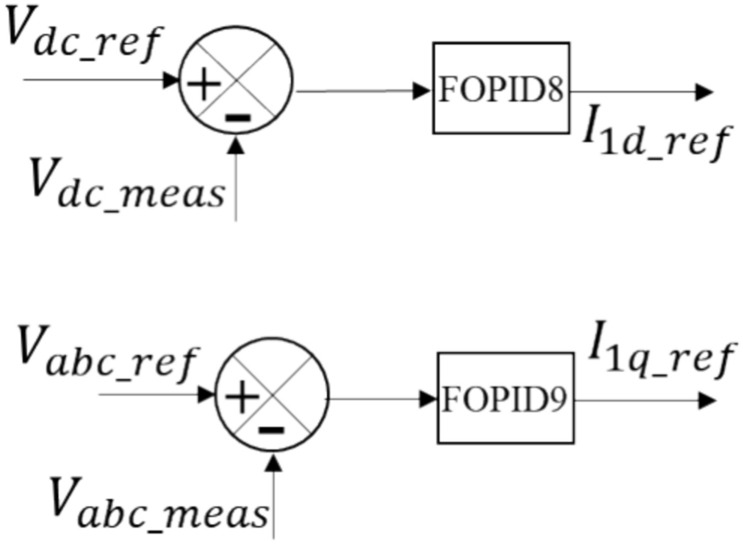
Outer voltage control setup.

In the HVDC transmission system, there are two VSC stations. The control methods used for both the offshore and onshore VSC stations are essential for the smooth functioning of the HVDC transmission system that employs VSC technology to connect an offshore wind farm to the onshore grid. The offshore VSC station is controlled using the cascaded control. The onshore VSC's internal current control loops are illustrated in Fig. 4. Figure 5 provides a closer view of the outer voltage control loop. The sending end VSC station is connected to the wind farm through the AC bus and has four control circuits. Equations (11) and (12) are utilized to calculate the voltage at the offshore VSC, which is controlled by the VSC^38^.

11 $${V}_{1d\_ref}={U}_{d}+\omega {L}_{2}{I}_{1q}-\left({R}_{2}{I}_{1d}+{L}_{2}\frac{d{I}_{1d}}{dt}\right)$$

12 $${V}_{1q\_ref}={U}_{q}-\omega {L}_{2}{I}_{1d}-\left({R}_{2}{I}_{1q}+{L}_{2}\frac{d{I}_{1q}}{dt}\right)$$

where the voltage components required at the VSC terminals are represented by ${V}_{1d\_ref}$ and ${V}_{1q\_ref}$ in the dq-axes. The transformer and reactor's resistance and inductance are denoted by ${R}_{2}$ and ${L}_{2}$, respectively. The current from the VSC has two dq-axes components: ${I}_{1\text{d}}$ and ${I}_{1q}$. Similarly, the voltage at PCC1 has two dq-axes components: ${U}_{d}$ and ${U}_{q}$. Equations (13) and (14) demonstrate how the FOPID6 and FOPID7 controllers drive two internal loops that control the currents ${I}_{1d}$ and ${I}_{1q}$, respectively, using the parameter gains ${k}_{p6}$, ${k}_{i6}$, ${k}_{d6}$, ${\alpha }_{6}$, ${\mu }_{6}$, ${k}_{p7}$, ${k}_{i7}$, ${k}_{d7}$, ${\alpha }_{7}$, and ${\mu }_{7}$. The outer loops consist of the parameter gains of the FOPID8 controller, ${k}_{p8}$,${k}_{i8}$, ${k}_{d8}$, ${\alpha }_{8}$, and ${\mu }_{8}$, which regulate the DC voltage using Eq. (15), and the parameter gains of the FOPID9 controller, ${k}_{p9}$, ${k}_{i9}$, ${k}_{d9}$, ${\alpha }_{9}$, and ${\mu }_{9}$, which regulate the AC voltage using Eq. (16).

13 $$\left({R}_{2}{I}_{1d}+{L}_{2}\frac{d{I}_{1d}}{dt}\right)={k}_{p6}\left({I}_{1dref}-{I}_{1d}\right)+{k}_{i6}{\left(\int \left({I}_{1dref}-{I}_{1d}\right)dt\right)}^{{\alpha }_{6}}+{k}_{d6}\frac{{d}^{{\mu }_{6}}\left({I}_{1dref}-{I}_{1d}\right)}{{dt}^{{\mu }_{6}}}$$

14 $$\left({R}_{2}{I}_{1q}+{L}_{2}\frac{d{I}_{1q}}{dt}\right)={k}_{p7}\left({I}_{1qref}-{I}_{1q}\right)+{k}_{i7}{\left(\int \left({I}_{1qref}-{I}_{1q}\right)dt\right)}^{{\alpha }_{7}}+{k}_{d7}\frac{{d}^{{\mu }_{7}}\left({I}_{1qref}-{I}_{1q}\right)}{{dt}^{{\mu }_{7}}}$$

15 $${I}_{1dref}={k}_{p8}\left({V}_{dc,ref}-{V}_{dc\_meas}\right)+{k}_{i8}{\left(\int \left({V}_{dc,ref}-{V}_{dc\_meas}\right)dt\right)}^{{\alpha }_{8}}+{k}_{d8}\frac{{d}^{{\mu }_{8}}\left({V}_{dc,ref}-{V}_{dc\_meas}\right)}{{dt}^{{\mu }_{8}}}$$

16 $${I}_{1qref}={k}_{p9}\left({V}_{pcc,ref}-{V}_{abc\_meas}\right)+{k}_{i9}{\left(\int \left({V}_{pcc,ref}-{V}_{abc\_meas}\right)dt\right)}^{{\alpha }_{9}}+{k}_{d9}\frac{{d}^{{\mu }_{9}}\left({V}_{pcc,ref}-{V}_{abc\_meas}\right)}{{dt}^{{\mu }_{9}}}$$

More information on the offshore VSC's four control loops is provided below: ${I}_{1d}$ represents the actual d-axis current monitored in the inner current control system depicted in Fig. 4. In the meantime, its reference value, ${I}_{1dref}$, is established by the outside voltage control loop. FOPID6 receives the difference as input. The voltage reference for the d-axis that will be utilized to regulate the voltage of the offshore VSC station is ${V}_{1d\_ref}$. ${I}_{1q}$ denotes the real q-axis current. Its reference value, ${I}_{1qref}$, is ascertained from the outside voltage control loop. FOPID7 receives the error. Therefore, the offshore VSC station's q-axis voltage reference is represented by the final output, ${V}_{1q\_ref}$.

Figure 5 illustrates the setup for the outer voltage control. It comprises two control loops: the DC voltage control loop and the AC voltage control loop. The DC voltage control loop regulates the targeted DC voltage level for the DC link, denoted as ${V}_{dc,ref}$, which is set to the rated per-unit voltage value. The actual measured DC link voltage is represented by ${V}_{dc\_meas}$, and any discrepancies between ${V}_{dc,ref}$ and ${V}_{dc\_meas}$ are processed by the FOPID8 controller. The output of the controller, ${I}_{1dref}$, is used to regulate the AC voltage at the PCC1, represented by ${V}_{pcc,ref}$, in relation to the AC voltage control loop. The real value for the AC voltage at PCC1, measured as ${V}_{abc\_meas}$, is processed by FOPID9, which produces ${I}_{1qref}$ as its output.

The HVDC transmission system has two converter stations—the offshore VSC station and the onshore VSC station. The onshore VSC station is also known as the receiving end converter. Both the offshore and onshore VSC stations have a similar control method. The FOPID10 and FOPID11 controllers use the parameters: ${k}_{p10}$, ${k}_{i10}$, ${k}_{d10}$, ${\alpha }_{10}$, ${\mu }_{10}$, ${k}_{p11}$, ${k}_{i11}$, ${k}_{d11}$, ${\alpha }_{11}$, and ${\mu }_{11}$ to drive two internal loops. The outer loops also include the parameter gains of FOPID12 controller, which are ${k}_{p12}$, ${k}_{i12}$, ${k}_{d12}$, ${\alpha }_{12}$, and ${\mu }_{12}$, responsible for controlling the DC voltage, and the parameter gains of FOPID13 controller, which are ${k}_{p13}$, ${k}_{i13}$, ${k}_{d13}$, ${\alpha }_{13}$, and ${\mu }_{13}$, responsible for regulating the AC voltage.

In this research, the cost function is defined by Eq. (17). This function is the integral square errors (ISE) of the RMS voltage at PCC2 on the onshore side. The nonlinear nature of the simulation model explains the use of this approach.

17 $$Fitness=\sum ISE=\int {\left({V}_{pcc,ref}-{V}_{pcc}\right)}^{2}dt$$

The following section presents a detailed explanation of the steps required to develop the WaOA technique for modifying the parameters of the FOPID controller.

## The proposed optimization algorithm

The WaOA is a new algorithm inspired by the behavior of walruses in nature. It mimics their social structures and skills to achieve effective optimization^34^. Walruses have a keen sense of touch, which the WaOA algorithm incorporates through population modeling and their reactions to "danger" and "safety" signals. The algorithm also considers the social structures and hierarchies that exist among adult, juvenile, and female walruses in their communities. These elements guide the WaOA's search strategy, balancing exploration and exploitation. This section explains the main concepts of the WaOA algorithm.

The WaOA optimization process begins by generating a first population of candidate solutions, which are randomly created to fall within the pre-established upper and lower constraints for the design variables of the optimization problem. This diverse starting point ensures that there is enough exploration of the search space for potential optimal solutions. Throughout the WaOA iterations, these agents move in response to social and environmental signals, constantly focusing on the best possible solutions.

The WaOA algorithm imitates how walruses respond to their environment by using "safety" and "danger" signals. These signals influence the behavior of every agent, which in turn guides the population towards areas where the best solutions are likely to be found. The danger signal reflects the risk associated with an agent's current position, and its calculation is based on Eq. (18)^34^. As optimization progresses, this risk signal gradually weakens, encouraging agents to move towards viable solutions. Equation (22) computes the safety signal which indicates the attractiveness of an agent's current location and gets stronger with every iteration, encouraging exploitation. The WaOA algorithm balances these pressures effectively to navigate the search space.

18 $$\text{Danger}\_\text{signal }= A\times R$$

19 $$A=2\times \alpha $$

20 $$\alpha =1-\frac{t}{T}$$

21 $$R=2\times {r}_{1}-1$$

22 $$\text{Safety}\_\text{signal}={r}_{2}$$

There are two risk factors denoted by $A$ and $R$. The parameter $\alpha$ decreases from 1 to 0 during the optimization process. The safety signal is indicated by ${r}_{2}$, while the other two stochastic variables, ${r}_{1}$ and ${r}_{2}$, fall between 0 and 1. The variable $t$ indicates the current iteration step, while $T$ represents the maximum predefined number of iterations.

During the migration stage, the walrus agents attempt to explore new areas of the search space. This is achieved by rearranging the position of each agent using a random integer ${r}_{3}$ and a migration step $\beta$. The following equation represents the walrus position update during this phase^34^:

23 $${X}_{ij}^{t+1}={X}_{ij}^{t}+Migration\_step$$

24 $$Migration\_step=\left({X}_{m}^{t}-{X}_{n}^{t}\right)\times \beta \times {r}_{3}^{2}$$

25 $$\beta =1-\frac{1}{1+{e}^{\frac{-10\left(t-0.5T\right)}{T}}}$$

where the modified location in iteration $i$ along dimension $j$ is represented by ${X}_{ij}^{t+1}$. Two randomly selected places are indicated by ${X}_{m}^{t}$ and ${X}_{n}^{t}$.

The stage of population diversification is called the Reproduction Stage. In this stage, three groups of walruses—males, females, and juveniles—exhibit different behaviors. Male walruses act as scouts and explore new areas within the search area. Their placements are modified as needed. Female walruses focus on refining potential ideas through exploitation. Young walruses bring even more variation to the population, and their placements are adjusted in response to their interactions with both parents. The aspects of exploitation and exploration are included in their behavior, and unpredictability is added to allow for further exploration.

The WaOA algorithm's ability to find optimal solutions is enhanced by striking a balance between exploring new regions and exploiting promising solutions using various reproduction techniques. Equations (26)–(28) provide the formulation for this stage^34^.

26 $${female}_{ij}^{t+1}={female}_{ij}^{t}+\alpha \times \left({male}_{ij}^{t}-{female}_{ij}^{t}\right)+\left(1-\alpha \right)\times \left({X}_{best}^{t}-{female}_{ij}^{t}\right)$$

27 $${Juvenile}_{ij}^{t+1}=\left(O-{Juvenile}_{ij}^{t}\right)\times P$$

28 $$O={X}_{best}^{t}+{Juvenile}_{ij}^{t}\times LF$$

Let $P$ represent the risk factor for young walruses, and $O$ represent the safety benchmark for their position.

## Simulation results

This section on the simulation results analyzes how well the system performs when faced with different fault scenarios. This is an essential aspect of the system’s ability to respond quickly and with minimal impact from fluctuations while remaining stable and connected to the grid during various fault scenarios. The onshore VSC station could encounter various fault situations, which are thoroughly investigated in this report. The study examines 13 controllers, including 5 for the OWFs, 4 for the onshore VSC, and an additional 4 for the offshore VSC. The optimization of the controllers is not performed simultaneously for all 13 controllers because this resulted in poor dynamic response. The study investigates the controller in the onshore VSC station which controls the AC voltage at PCC2. The gains of other controllers can be found in^41^.

Based on the simulation results, it has been observed that the suggested WaOA technique is more efficient than meta-heuristic approaches when it comes to optimizing the gains of the FOPID controller in different fault scenarios. The main objective of this optimization strategy is to improve the FRT capabilities of the OWF under various fault scenarios. Equation (10) has been used as a fitness function to achieve this goal. The number of iterations is set to 10. The number of populations is specified as 5. There are constraints on the controller gains, with the minimum limit being 0 and the maximum limit for ${k}_{p}$, ${k}_{i}$, ${k}_{d}$ set at 100. Additionally, the alpha and mu parameters have their own specified limits, which range from 0 to 2. The simulation results validated the accuracy of our model. Fine-tuning the converter’s FOPID controllers using the WaOA technique resulted in notable improvements. Tables 3 and 4 present a comprehensive evaluation of controller parameters and gains, respectively. Table 3 outlines the parameters of the FOPID controller obtained through the proposed WaOA technique, while Table 4 details the gains of the PI controller optimized by both PSO and GA for the same system structure except for the controller type, demonstrating the superior performance of WaOA over these techniques.

**Table 3:** Optimized FOPID controller parameters for the proposed WaOA.

		${k}_{p}$	${k}_{i}$	${k}_{d}$	$\alpha$	$\mu$
Wind station	1	7.32	26.33	2.23	0.8	0.8
	2	7.32	26.33	2.23	0.8	0.8
	3	0.7	2.4	0.11	0.112	0.92
	4	17.33	18.24	0.2	0.88	0.98
	5	17.33	18.24	0.2	0.88	0.98
Offshore station	6	12.14	60.22	0.29	0.298	0.89
	7	27.52	6.9	0.51	0.83	0.93
	8	3.75	30.44	0.11	0.118	0.934
	9	3.75	30.44	0.11	0.118	0.934
Onshore station	10	6.78	100	0.42	0.423	0.88
	11	2.1	0.54	0.69	0.9	0.9
	12	12.77	48.23	0.19	0.194	0.972
	13	2.6	14	0.19	0.9	0.9

**Table 4:** Optimized PI controller parameters for GA and PSO.

		PSO		GA	
		${k}_{p}$	${k}_{i}$	${k}_{p}$	${k}_{i}$
Wind station	1	5.2557	24.3747	15.6187	5.6171
	2	5.2557	24.3747	15.6187	5.6171
	3	0.3986	2.9042	0.858	1.959
	4	67.1374	87.8442	74.31	82.846
	5	67.1374	87.8442	74.31	82.846
Offshore station	6	2.7042	15.3277	0.589	87.937
	7	2.7042	15.3277	0.589	87.937
	8	1.846	35.8433	13.835	18.179
	9	8.0622	96.9977	1.587	9.689
Onshore station	10	0.587	14.9644	37.828	61.81
	11	0.587	14.9644	37.828	61.81
	12	25.1128	41.1542	8.955	7.995
	13	2.1779	0	1.6	10

### Scenario 1: three-phase to ground fault

**Figure 6:**
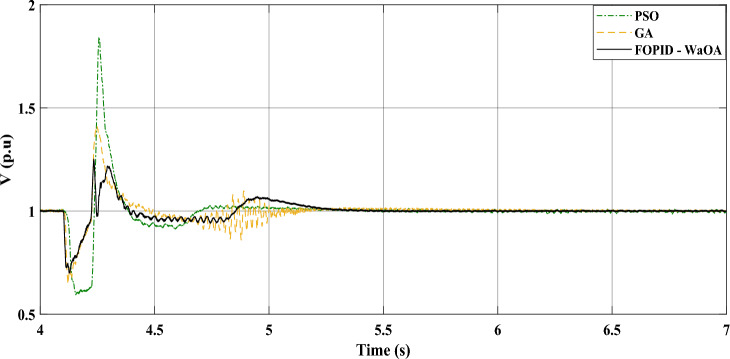
${V}_{dc}$ response to a symmetrical fault.

**Figure 7:**
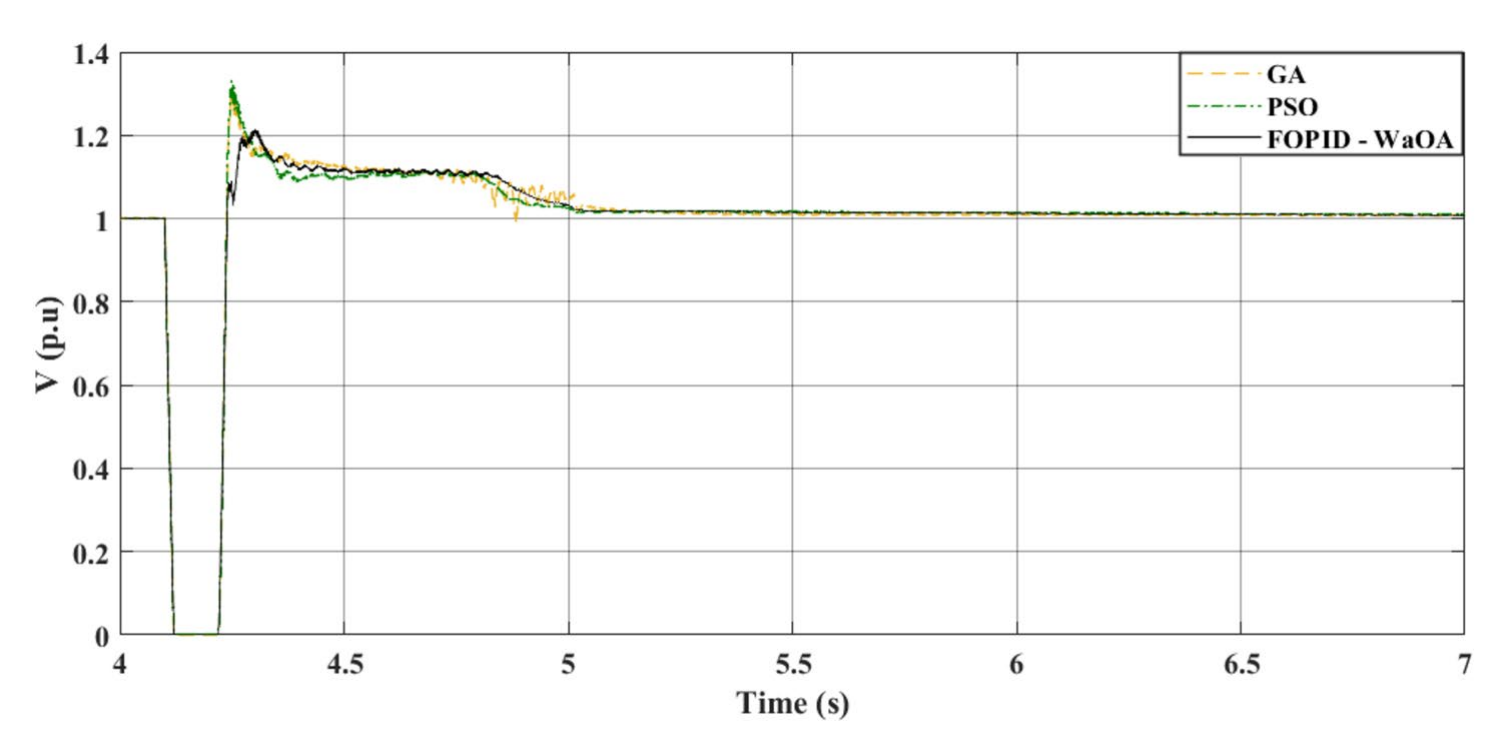
${V}_{ac}$ at onshore VSC (symmetrical fault).

**Figure 8:**
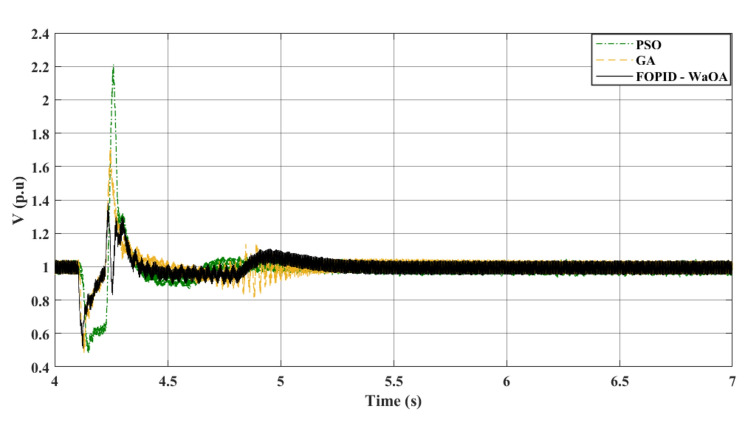
${V}_{ac}$ at offshore VSC (symmetrical fault).

In the studied system whose controller is optimized by the WaOA, a 3-phase symmetrical fault scenario was simulated. It is the first scenario in this study. The WaOA’s performance was compared to algorithms; GA, and PSO. The points of comparison between the presented optimization algorithms are the settling time, the steady state error, the maximum peak overshoots, and undershoots. The simulation, at a wind speed of 12 m/s, took approximately 7 s to reach steady state. That is why the simulation time is set to 7 s. A three-phase fault was introduced at location F at 4.1 s (refer to Fig. 1). This location is the point of common coupling 2. This is the point of interconnection between the WECS and the grid. At this point, there are two parallel lines. The faulty line circuit breaker opened at 4.22 s and reclosed at 5 s. This means that the time taken by the circuits breaker to operate, the fault duration, is 0.12 s. The fault is assumed to be cleared upon the first reclosure. The DC and terminal AC voltages are shown in Figs. 6 and 7, respectively. Meanwhile, Fig. 8 focuses on the AC voltage on the offshore side. The simulation results indicated that WaOA surpassed other algorithms by achieving faster settling times, experiencing less fluctuation, and exhibiting a shorter peak overshoot in the voltage profiles. This translates to improved system stability during fault events.

Comparing the transient performance can provide useful insights. When it comes to improving the dynamic response of a system, WaOA performs better than both PSO and GA. This is demonstrated by the shorter maximum overshoots in AC voltage, which indicate minor fluctuations and quicker settling times. In the case of a symmetrical fault scenario, WaOA can help ${V}_{dc}$ and ${V}_{ac}$ return to their pre-fault levels swiftly and with minimal fluctuation, overshoot, or undershoot. Overall, the most effective choice for improving the transient behavior of both ${V}_{dc}$ and ${V}_{ac}$ in the symmetrical fault scenario is WaOA.

### Scenario 2: line to ground fault

In the second scenario, a LG fault incidence is simulated at 4.1 s. The location of the fault in the second scenario is the same for the fault location in the first scenario. The simulation lasts for 7 s, as the system reaches steady state. Throughout this event, the CB operates at 4.22 s and closes again at 5 s. The characteristics of the wind system remain the same as in the preceding case. When WaOA is used to optimize FOPID gains, remarkable improvements in ${V}_{dc}$ and ${V}_{ac}$ performance are demonstrated. In this study, the optimization is done on the most severe fault scenario, the symmetrical fault. After that, the gains obtained in the most severe scenario is used for verification in the second scenario.

**Figure 9:**
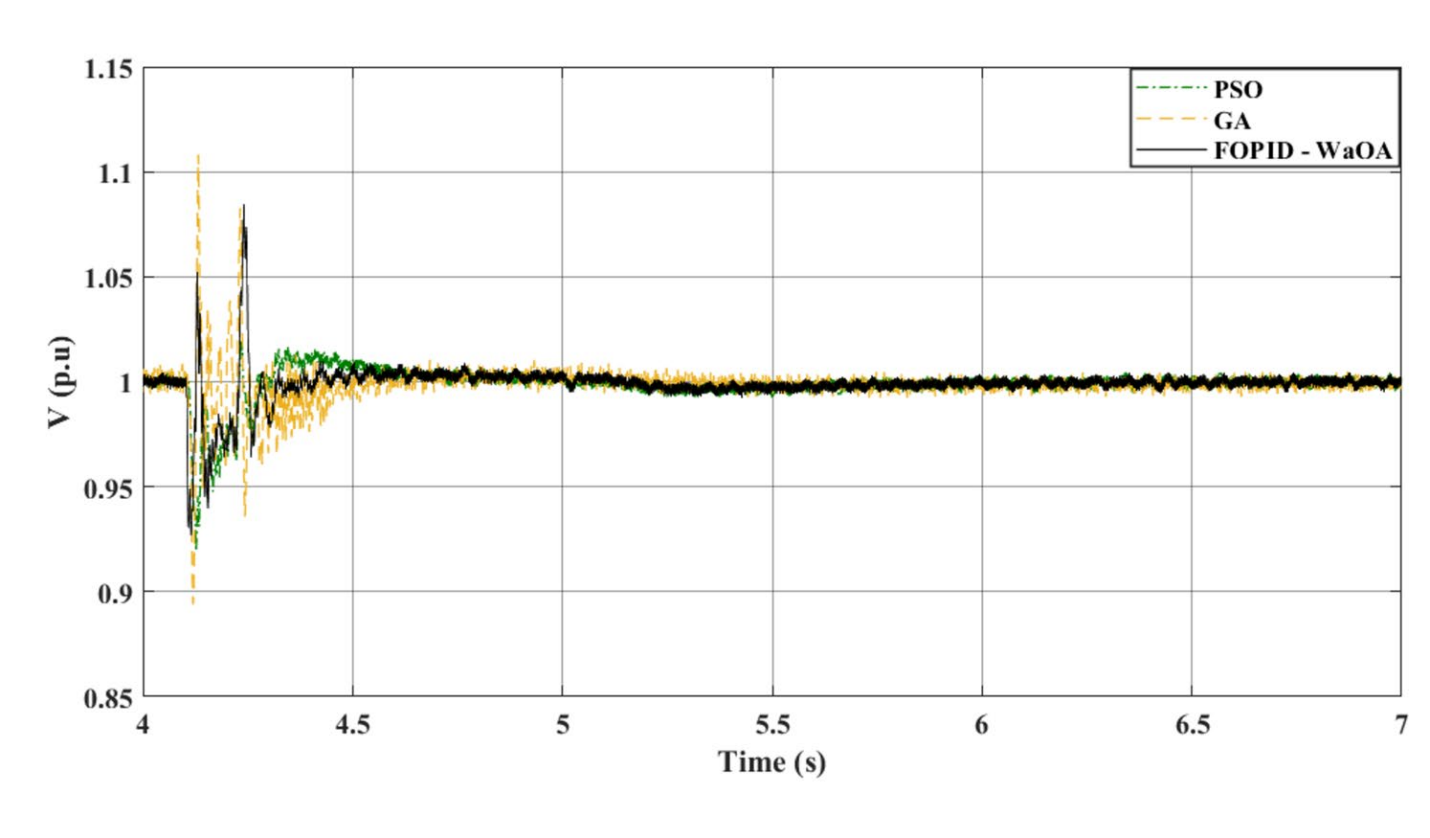
${V}_{dc}$ response to a L-G fault.

**Figure 10:**
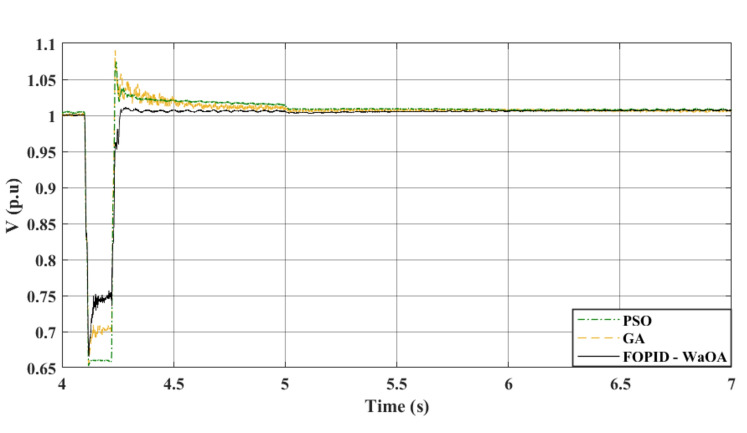
${V}_{ac}$ at onshore VSC (L-G fault).

**Figure 11:**
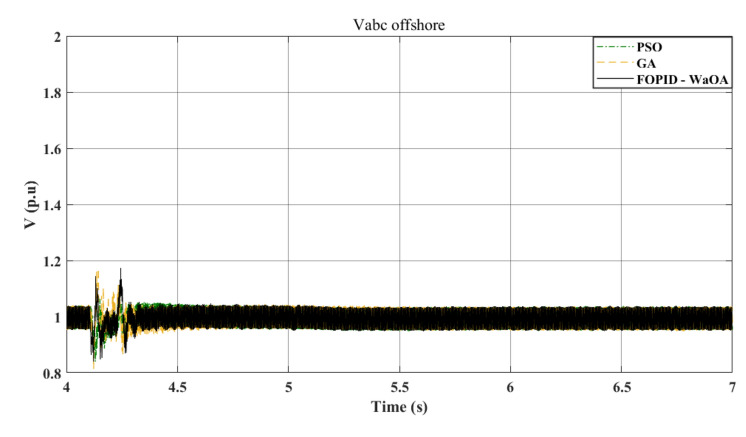
${V}_{ac}$ at offshore VSC (L-G fault).

Figure 9 shows that the proposed WaOA algorithm performs better than PSO and GA in terms of improving ${V}_{dc}$ settling time, maximum peak over and undershoots. Additionally, Fig. 10 demonstrates that the introduction of WaOA enhances the transient stability of the AC voltage at the onshore VSC compared to PSO and GA. WaOA outperforms other optimization methods due to its minimum over shoots and undershoots, low steady-state error, and low oscillations, which help ${V}_{dc}$ and ${V}_{ac}$ return to their pre-fault levels quickly. Moreover, Fig. 11 provides an overview of the AC voltage at the offshore VSC throughout the simulation. The AC voltage profiles at the offshore VSC show that the performance of the three presented algorithms are close to each other in terms of settling times, maximum peak over and undershoots.

### Scenario 3: phase-to- phase fault

**Figure 12:**
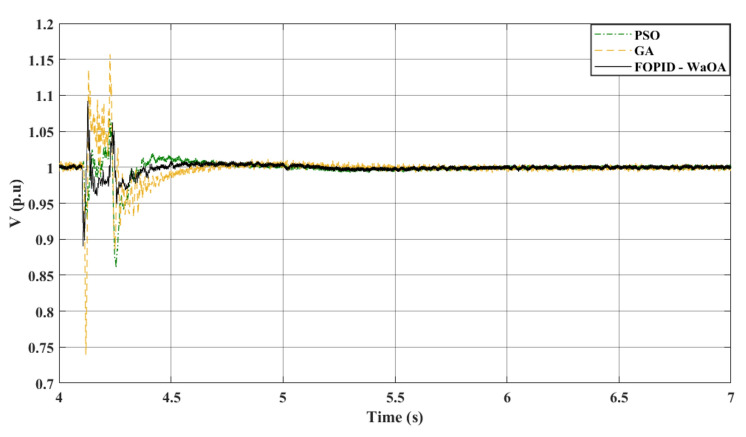
${V}_{dc}$ response to a L-L fault.

**Figure 13:**
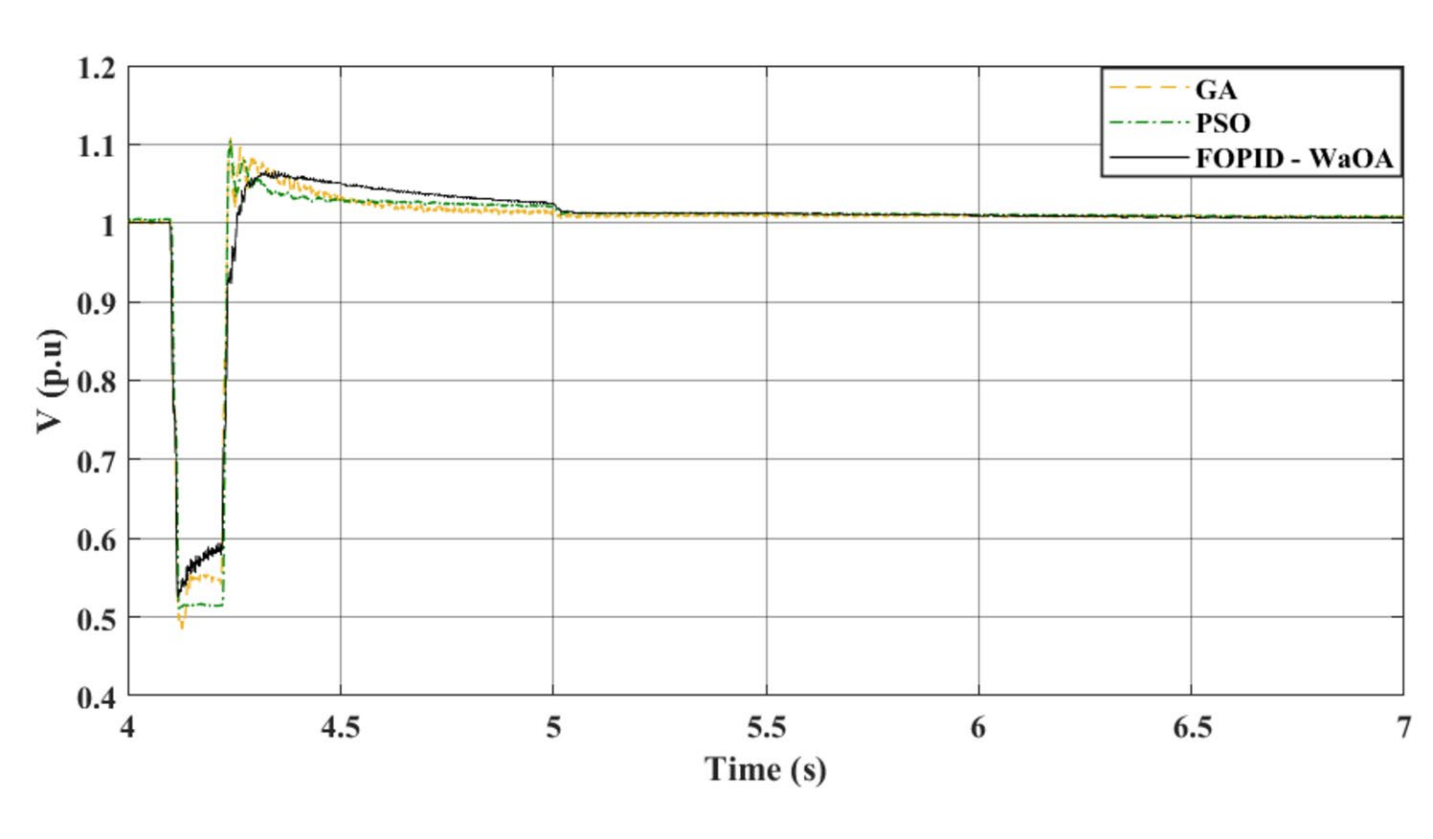
${V}_{ac}$ at onshore VSC (L-L fault).

**Figure 14:**
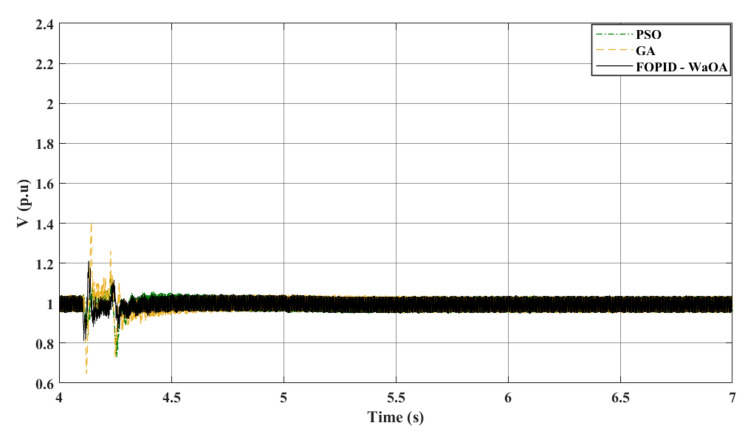
${V}_{ac}$ at offshore VSC (L-L fault).

In the third scenario, the wind farm’s parameters remain constant, however, an LL fault is introduced to examine the system’s response to a new scenario. At 4.1 s, the fault occurs, and the circuit breaker opens at 4.22 s, closing again at 5 s. The gains of the controllers set in this scenario are the same gains obtained by the optimization methods in the first and most severe fault scenario. When applying these gains and comparing the tuning of the FOPID controller with other metaheuristics, the advantages of WaOA become apparent. WaOA enhances system efficiency by reducing peak overshoot, settling time, and fluctuations. Figure 12 confirms these enhancements, demonstrating ${V}_{dc}$. Figure 13 presents the AC voltage at the onshore VSC. It is shown that the AC voltage profile at the onshore VSC station obtained by the proposed WaOA is better in maximum peak over and undershoots than the other optimization methods. Meanwhile, the AC voltage at the offshore VSC is shown in Fig. 14. It is observed that the voltage profile reflects that the response of the proposed WaOA optimization is better than the GA. Meanwhile, the performance of the WaOA and the PSO are close to each other.

### Scenario 4: double line to ground fault

**Figure 15:**
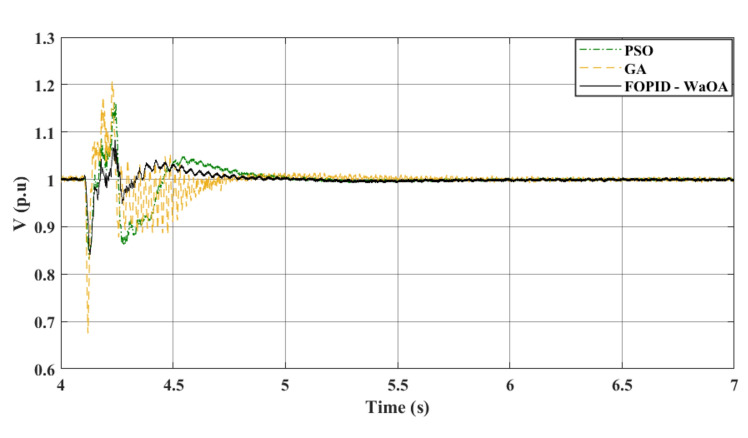
${V}_{dc}$ response to a L-L-G fault.

**Figure 16:**
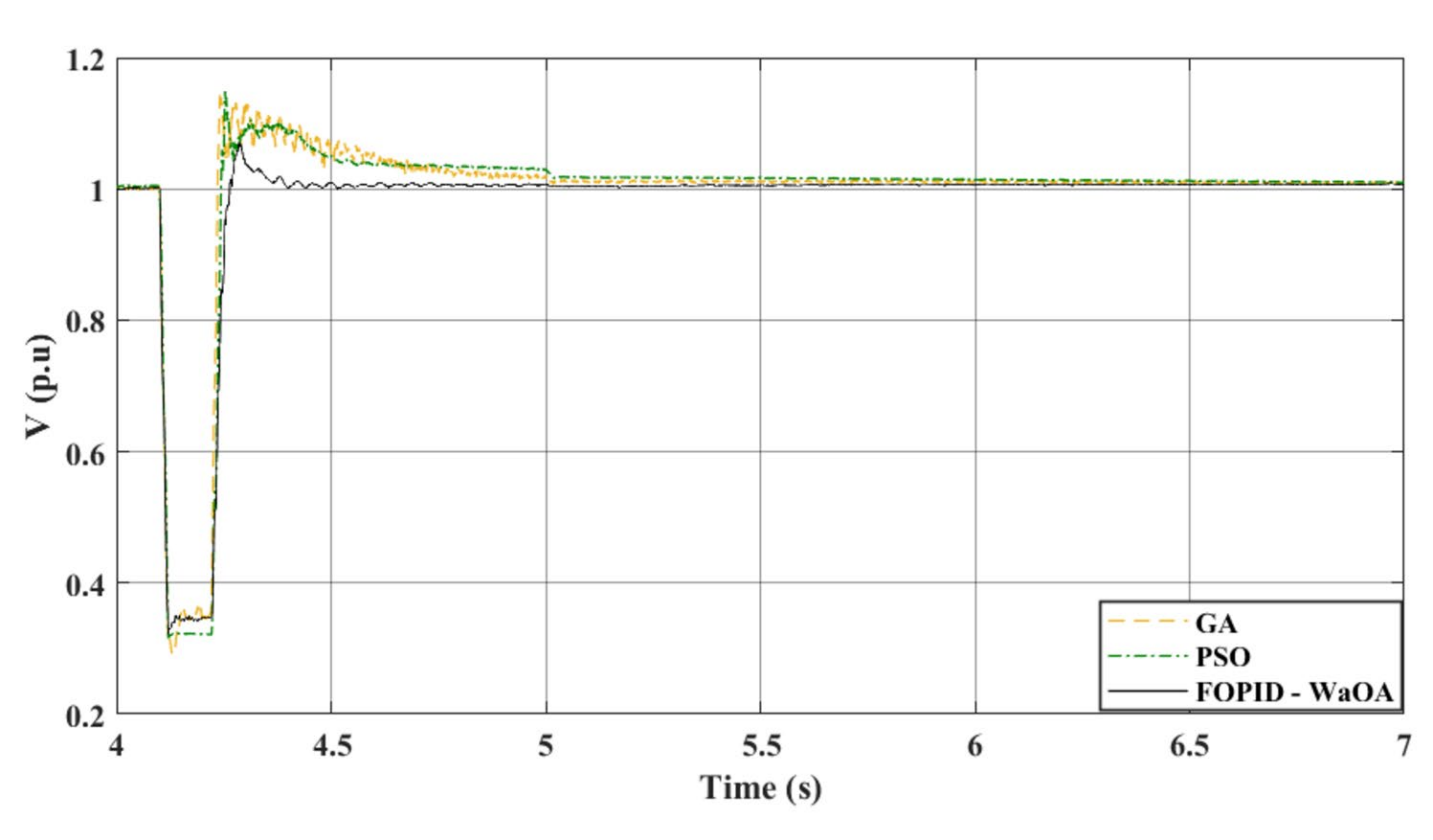
${V}_{ac}$ at onshore VSC (L-L-G fault).

**Figure 17:**
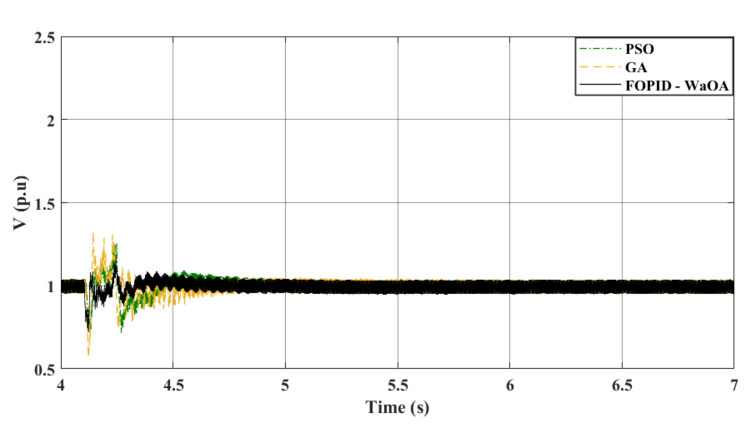
${V}_{ac}$ at offshore VSC (L-L-G fault).

In the fourth and final scenario, the system experienced a LLG fault at 4.1 s at the same fault location of the previous three scenarios. The efficiency of WaOA in optimizing the controller gains and obtaining better dynamic response was compared to GA and PSO in terms of system disturbances caused by faults. The results are presented in Figs. 15 and 16, which show the DC voltage profile and the AC voltage profile at the onshore VSC station side, respectively. These two figures demonstrate the superiority of WaOA in reducing oscillations, peak overshoot, and settling time. Also, Fig. 17 shows the AC voltage at the offshore VSC station during the simulation. It is observed from the AC voltage profiles at the offshore VSC station that the WaOA performed better than the other optimization algorithms in terms of maximum peak overshoots and undershoots as well as settling time. Overall, the proposed WaOA metaheuristic algorithm proved to be highly effective in improving the performance of offshore wind turbines, particularly in various fault scenarios.

In the following table, Table 5, a comparative analysis of the MPOS and MPUS of AC voltage profiles at an onshore VSC station for various fault types using WaOA, GA, and PSO is presented.

**Table 5:** Comparative analysis of MPOS and MPUS of AC voltage profiles at onshore VSC station for different fault types using WaOA, GA, and PSO.

Fault type	Point of comparison	WaOA	GA	PSO
3L-G	Max. overshoot (p.u)	0.215	0.3171	0.3323
	Min. undershoot (p.u)	− 1	− 1	− 1
L-G	Max. overshoot (p.u)	0.01071	0.09034	0.0743
	Min. undershoot (p.u)	− 0.3346	− 0.3452	− 0.3478
L-L	Max. overshoot (p.u)	0.0649	0.11045	0.10507
	Min. undershoot (p.u)	− 0.4746	− 0.50523	− 0.491773
L-L-G	Max. overshoot (p.u)	0.07164	0.14736	0.14872
	Min. undershoot (p.u)	− 0.677	− 0.7087	− 0.6837

## Conclusions

This paper presented a novel design approach for optimizing the gains of FOPID controllers in VSC stations of offshore wind energy systems. The approach utilizes the WaOA, a recently published metaheuristic technique inspired by the foraging behavior of walruses. This study contributes to the field in three keyways. First, it validates the effectiveness of the WaOA in optimizing FOPID controller gains for VSC control in offshore wind energy systems. Second, it investigates the system's dynamic response under various fault scenarios, including symmetrical faults, L-G faults, L-L faults, and L-L-G faults. Third, it conducts a comparative analysis between FOPID controllers with gains optimized using the WaOA and PI controllers optimized by other established algorithms like PSO and GA across all diverse fault types. The results demonstrate the effectiveness of the WaOA in optimizing the VSC station controller. It was observed that WaOA-tuned controllers consistently achieved improved system dynamic response, as measured by metrics like voltage peak overshoots, undershoots, and settling time. Furthermore, controllers optimized with the WaOA outperformed those optimized with other algorithms across various fault scenarios, including both symmetrical and unsymmetrical faults. In case of symm. fault, using WaOA enhanced the MPOS by up to 32% in the DC link voltage, and by up to 9% in the AC voltage at the onshore VSC station, and by up to 38% in the AC voltage at the offshore VSC station, over the other optimization methods GA and PSO. Also, WaOA enhanced the MPUS by up to 18% in the DC voltage, and by up to 8% at the AC voltage at the offshore VSC station. In case of L-G fault, using WaOA enhanced the MPOS by up to 7% in the AC voltage at the onshore VSC station. Also, WaOA enhanced the MPUS by up to 4% in the DC voltage, and 2% in the AC voltage at the onshore VSC station. In case of L-L fault, using WaOA enhanced the MPUS by up to 20% in the DC voltage, and 6% in the AC voltage at the onshore VSC station. In case of L-L-G fault, using WaOA enhanced the MPOS by up to 10% in the DC voltage, and 7% in the AC voltage at the onshore VSC station. Also, WaOA enhanced the MPUS by up to 25% in the DC voltage, and 11% in the AC voltage at the onshore VSC station. This superior performance suggests that the WaOA has the potential to be a powerful tool for enhancing system stability. FOPID controllers with WaOA-optimized gains achieved faster post-fault recovery, leading to an overall improvement in system dependability. The robustness of the WaOA approach to diverse disturbances, as demonstrated through simulations. As a results, it is recommended to employ the WaOA as a powerful optimization tool in further optimization problem in the field of power systems including renewable energy sources integration, as a future work and extension to this research study. The success of the WaOA in optimizing FOPID controller gains for VSC control in this study suggests its potential as a powerful tool for future research on optimization problems in power systems, particularly those involving renewable energy source integration. Moreover, future work can also focus on experimental validation using embedded systems to further verify the effectiveness of the proposed approach.

## Data Availability

The datasets used and/or analysed during the current study available from the corresponding author on reasonable request.
